# The Long Non-Coding Antisense RNA JHDM1D-AS1 Regulates Inflammatory Responses in Human Monocytes

**DOI:** 10.3389/fcimb.2022.934313

**Published:** 2022-07-12

**Authors:** Erik Malmström, Hina N. Khan, Cornelis van ‘t Veer, Melissa Stunnenberg, Mariska T. Meijer, Hisatake Matsumoto, Natasja A. Otto, Teunis B. H. Geijtenbeek, Alex F. de Vos, Tom van der Poll, Brendon P. Scicluna

**Affiliations:** ^1^ Amsterdam University Medical Centers, Center for Experimental and Molecular Medicine, University of Amsterdam, Amsterdam, Netherlands; ^2^ Division of Infection Medicine, Department of Clinical Sciences, Lund University, Lund, Sweden; ^3^ Emergency Medicine, Department of Clinical Sciences Lund, Lund University, Skane University Hospital, Lund, Sweden; ^4^ Amsterdam University Medical Centers, Clinical Epidemiology and Data Science, University of Amsterdam, Amsterdam, Netherlands; ^5^ Amsterdam Institute for Infection and Immunity, Amsterdam, Netherlands; ^6^ Amsterdam University Medical Centers, Experimental Immunology, University of Amsterdam, Amsterdam, Netherlands; ^7^ Amsterdam University Medical Centers, Division of Infectious Diseases, University of Amsterdam and Vrije Universiteit Amsterdam, Amsterdam, Netherlands; ^8^ Department of Applied Biomedical Science, Faculty of Health Sciences, Mater Dei hospital, University of Malta, Msida, Malta; ^9^ Centre for Molecular Medicine and Biobanking, University of Malta, Msida, Malta

**Keywords:** long non-coding RNA, monocyte, toll-like receptors, sepsis, inflammation

## Abstract

Monocytes are key players in innate immunity, with their ability to regulate inflammatory responses and combat invading pathogens. There is a growing body of evidence indicating that long non-coding RNA (lncRNA) participate in various cellular biological processes, including the innate immune response. The immunoregulatory properties of numerous lncRNAs discovered in monocytes remain largely unexplored. Here, by RNA sequencing, we identified a lncRNA JHDM1D-AS1, which was upregulated in blood monocytes obtained from patients with sepsis relative to healthy controls. *JHDM1D-AS1* expression was induced in primary human monocytes exposed to Toll-like receptor ligands, such as lipopolysaccharide (LPS), or bacteria. The inducibility of *JHDM1D-AS1* expression in monocytes depended, at least in part, on nuclear factor–κB activation. JHDM1D-AS1 knockdown experiments in human monocyte-derived macrophages revealed significantly enhanced expression of inflammatory mediators, before and after exposure to LPS, relative to control cells. Specifically, genes involved in inflammatory responses were upregulated (e.g., *CXCL2*, *CXCL8*, *IL1RN*, *TREM1*, *TNF*, and *IL6*), whereas genes involved in anti-inflammatory pathways were downregulated (e.g., *SOCS1* and *IL10RA*). *JHDM1D-AS1* overexpression in a pro-monocytic cell line revealed diminished pro-inflammatory responses subsequent to LPS challenge. Collectively, these findings identify *JHDM1D-AS1* as a potential anti-inflammatory mediator induced in response to inflammatory stimuli.

## Introduction

Monocytes play an essential role in orchestrating the inflammatory response, with the ability to mount complex effector functions, including pathogen recognition, phagocytosis, antigen presentation, and cytokine secretion (Ginhoux and Jung, 2014; Jakubzick et al., 2017). To adequately fulfill their function in innate immunity, monocytes utilize a vast array of pattern recognition receptors, such as Toll-like receptors (TLRs), that recognize pathogen-associated molecular patterns, and signal *via* intracellular signaling proteins, such as myeloid differentiation primary response gene 88 (MyD88), to activate the key transcription factor of inflammatory transcriptional responses nuclear factor–κB (NF-κB) (Medzhitov and Horng, 2009; Liu et al., 2017). Activation of NF-ĸB results in the production of pro- and anti-inflammatory cytokines that include tumor necrosis factor (TNF), interleukin-1β (IL-1β), IL-6, and IL-10. Cytokine gene expression and secretion is a tightly regulated process, and dysregulated cytokine signaling can lead to serious conditions such as sepsis (Poll et al., 2017).

Recent advances in next-generation sequencing have enabled the identification of several thousands of non-coding RNA molecules over the last couple of years, arbitrarily classified as long or small non-coding RNA based on nucleotide length >200 or <200, respectively (Ulitsky and Bartel, 2013; Necsulea and Kaessmann, 2014). Regions of the genome void of protein-coding genes have been shown to be actively transcribed in the context of various diseases (Esteller, 2011). Small non-coding RNAs, mainly microRNAs, and long non-coding RNAs (lncRNAs) were linked to specific immune processes (Fitzgerald and Caffrey, 2014; Mehta and Baltimore, 2016). While microRNAs have been established as epigenetic modifiers of gene expression, studies on the functional aspects of lncRNAs have only recently begun. For the vast majority of non-coding RNAs, particularly lncRNAs, the functional significance is as yet unclear (Palazzo and Lee, 2015). However, there is an increasing amount of evidence showing that lncRNAs possess important regulatory potential in a wide variety of biological processes, including the immune system (Heward and Lindsay, 2014; Atianand and Fitzgerald, 2014). For example, targeted deletion of the lncRNA *LUCAT1* in myeloid cells resulted in deregulated interferon responses by a mechanism that involved interactions with Signal transducer and activator of transcription 1 (STAT1), a master transcriptional regulator of interferon response genes (Agarwal et al., 2020). To date, a limited number of lncRNAs have been described as functional units of immune responses, including lncRNA-Cyclooxygenase-2 (COX2) (Carpenter et al., 2013), lncRNA-erythroid prosurvival (EPS) (Atianand et al., 2016) and Morrbid (Kotzin et al., 2019). Our group has recently mapped the vast landscape of lncRNAs in blood leukocytes of patients with sepsis, as well as healthy subjects intravenously injected with the bacterial agonist lipopolysaccharide (LPS), showing pervasive expression of numerous lncRNAs with potential pathophysiological implications (Scicluna et al., 2020).

In this study, we used RNA sequencing data of circulatory blood monocytes obtained from patients with sepsis to identify the elevated antisense lncRNA *JHMD1D-AS1*, relative to healthy participants. We subsequently demonstrated that, in both circulatory blood monocytes and monocyte-derived macrophages (MDMs) exposed to TLR-agonists, *JHMD1D-AS1* expression was induced in a temporal specific manner. Using small interfering RNA (siRNA) electroporation to render cells deficient of JHDM1D-AS1 and overexpression experiments, we revealed a role for lncRNA *JHDM1D-AS1* in cell development, as well as in restraining pro-inflammatory gene expression. Altogether, these findings identify antisense lncRNA *JHDM1D-AS1* as a potential repressor of the inflammatory response in human primary monocytes and macrophages.

## Materials and Methods

### Patient Samples

Blood samples were obtained from six patients with sepsis caused by community-acquired pneumonia within 24 h after admission to the intensive care unit of Amsterdam UMC, location Academic Medical Center (AMC), University of Amsterdam, The Netherlands, and from four healthy subjects (Khan et al., 2020). Sepsis was defined on the basis of a “probable” or “definite” infection according to the Center for Disease Control and Prevention four-point scale (Garner et al., 1988) combined with at least one of general, inflammatory, hemodynamic, organ dysfunction, or tissue perfusion parameters derived from the International Sepsis Forum consensus definitions (Calandra et al., 2005), as previously described in detail (Klouwenberg et al., 2013). Monocytes were isolated using fluorescence-activated cell sorting (FACS) and stored in RNA protect cell reagent (Qiagen, Hilden, Germany). Total monocytes were identified as CD14^+^CD15^−^ cells in morphologically defined gates and sorted on FACS Canto II flow cytometer (BD Biosciences) with anti-CD14 antibody (Miltenyi Biotech), as previously described (Hoogendijk et al., 2017; Khan et al., 2020). The Medical Ethics Committee of the Academic Medical Center approved the study (IRB no. 10-056C), and written informed consent was obtained from all patients (or legal representative) and healthy controls.

### Primary Monocyte and Macrophage Preparation

Healthy volunteers for blood sampling were recruited in accordance with a study protocol that was reviewed by the Academic Medical Center Medical Ethical Committee (no. 2015_074). Prior to sample donation, all donors gave informed consent. Heparinized blood, from healthy donors or buffy coat, was diluted (1:1) with phosphate-buffered saline (PBS) and peripheral blood mononuclear cells (PBMCs) were isolated by density-gradient centrifugation (1,700 RPM for 30 min at 21°C; acceleration, 1; breaks, 0) using Ficoll-Paque PLUS (GE healthcare). PBMCs were washed twice in cold PBS containing 0.5% sterile endotoxin-free bovine serum albumin (Divbio Science Europe). CD14^+^ human monocytes were further purified with an anti-CD14–coated microbead-based purification step as per the manufacturer’s instructions (Miltenyi Biotec, Bergisch Gladbach, Germany). CD14^+^ monocytes were either directly used in cell stimulation experiments described below or differentiated into MDMs.

For MDMs, CD14^+^ monocytes cells were seeded in six-well plates at a density of 1 × 10^6^ cells per well in RPMI 1640 (Gibco, Amarillo, TX) with 10% sterile fetal calf serum (FCS), gentamicin (10 µg/ml; Lonza Bioscience, Durham, NC), 2 mM GlutaMAX (Thermo Fisher Scientific, Waltham, MA), 1 mM sodium pyruvate (Thermo Fisher Scientific, Waltham, MA), recombinant human granulocyte macrophage-colony stimulating factor (5 ng/ml; Prospec, Ness-Ziona, Israel) and cultured for 7 days. The cell medium was changed, and fresh medium was added on days 3 and 5. On day 7, the cells were washed and harvested using Tryple-select (Thermo Fisher Scientific, Waltham, MA) and replated to appropriate cell concentration 24 h prior to stimulation or used directly for RNA interference experiment.

### Cell Stimulation

For stimulation experiments of CD14^+^ monocytes, cells were seeded in a 48-well plate (677970; Greiner Bio-One, Frickenhausen, Germany) at a density of 5 × 10^5^ cells per well in 400 µl of RPMI 1640 (Gibco) with 10% sterile FCS, gentamicin (10 µg/ml; Lonza Bioscience, Durham, NC), 2 mM GlutaMAX (Thermo Fisher Scientific, Waltham, MA), and 1 mM sodium pyruvate (Thermo Fisher Scientific, Waltham, MA*)*. Cells were subsequently stimulated with medium control, LPS (from *Escherichia coli* O111:B4; 100 ng/ml; *In vivo* gen, San Diego, CA), Pam3CSK4 (1 µg/ml; *In vivo* gen), flagellin (from *P. aeruginosa*; 1 µg/ml; FLA-PA Ultrapure, *In vivo* gen), and poly(I:C) High Molecular Weight (HMW) (10 µg/ml, *In vivo* gen) for 2 h or heat-killed bacteria [cell-bacteria ratio, 1:10; *Streptococcus* (*S*.) *pneumoniae* ATCC^®^ 6303 and *Klebsiella* (*K*.) *pneumoniae* ATCC43816] for 2, 6, and 24 h. Medium was collected, and the cells were stored in RNA protect cell reagent (Qiagen, Hilden, Germany) until further processing. In a separate experiment, primary monocytes (5 × 10^5^cells per well) were pretreated with the NF-κB inhibitor BAY11-7082 (Malladi et al., 2004) (10 µM; Tocris Bioscience, Bristol, UK) or vehicle control (dimethyl sulfoxide; Merck, Darmstadt, Germany) for 30 min and then incubated in medium, with or without LPS (100 ng/ml) for 2 h.

### RT-qPCR

Real-time quantitative RT-PCR (RT-qPCR) was performed to evaluate RNA expression. Total RNA was extracted using Nucleospin RNA isolation kits (Macherey-Nagel, Germany), according to the manufacturer’s protocol, and reverse-transcribed using Moloney murine leukemia virus reverse transcriptase with random hexamer primers or oligo(dT) primer (Promega, Madison, WI). RT-qPCR analysis was performed using the SensiFast Sybr green mix (Bioline, London, UK) on a LightCycler system (LC480, Roche Applied Science, Penzberg, Germany). LinRegPCR software was used to analyze the data (Untergasser et al., 2021). Primers used are shown in **Supplementary Table 1**.

### Enzyme-Linked Immunosorbent Assay

Human TNF, IL-6, and IL-10 protein levels were measured by enzyme-linked immunosorbent assay (ELISA) (all DuoSet R&D systems, Minneapolis, MN) according to the manufacturer’s instructions.

### RNA Interference

RNA interference was performed using the Neon Transfection System according to the manufacturer’s instruction (Thermo Fisher Scientific, Waltham, MA). MDMs were washed with PBS twice and resuspended in buffer R (Thermo Fisher Scientific, Waltham, MA) with either short interfering (si) SMARTpool RNAs against JHDM1D-AS1 (R-187292-00, Dharmacon, Lafayette, CO) or siNon-Target as a control (D-001206-13, Lafayette, CO). The cells were subsequently subjected to 1,500 V for 20 ms to achieve transfection of siRNA. Transfected cells were seeded in 24-well plates in RPMI 1640 with 10% FCS (Gibco, Amarillo, TX) and 2 mM L-glutamine (Lonza Bioscience, Durham, NC), without antibiotics. After 48 h, viable cells were harvested, washed, and replated at a cell density of 2.0 to 5 × 10^5^cells per well in a 48-well plate in RPMI 1640 (Gibco, Amarillo, TX) medium containing 10% FCS, penicillin (100 U/ml), and 2 mM L-glutamine. Seventy-two hours after transfection, fresh medium was added, and cells were subsequently treated with LPS (100 ng/ml) or medium control for 2 and 6 h. Silencing of JHDM1D-AS1 expression was confirmed by RT-qPCR.

### Retroviral Transfection of PLAT A Cells and Transduction of THP1-MD2-CD14 Cells

For retroviral transfection of PLAT A cells (Cell Biolabs Inc., San Diego, CA), 1.2 × 10^6^ cells per condition were plated in Advanced TC six-well plates (Greiner Bio‐One, Kremsmünster, Austria). The cells were incubated overnight at 37°C in the presence of transfection complexes containing 2 μg of PMX-puro retroviral vector (Cell Biolabs Inc., San Diago, CA) with or without full-length human JHDM1D-AS1 (synthesized by GenScript Biotech, Leiden), 0.4 μg of pCL‐Ampho (Novus Biologicals), and Lipofectamine 2000 (Thermo Fisher) supplemented Iscove's Modified Dulbecco's Medium (IMDM) medium (Thermo Fisher) containing 10% FCS. After overnight incubation, the supernatants of transfected cells were replaced with 1.5 ml of complete RPMI. Forty‐eight hours after transfection, viral supernatants were harvested, filtered over a 0.45-μm filter, and used to transduce THP1-MD2-CD14 cells (*In vivo* gen, San Diego, CA). PLAT A cells were washed with PBS and resuspended for FACS analysis to determine the transfection efficiency. For the transduction, 1 × 10^6^ THP1-MD2-CD14 cells in 1 ml of complete RPMI were seeded in a regular six-well plate. Virus-containing supernatant was mixed with polybrene (Sigma, Saint Louis, MO) before adding 1 ml to each well (final polybrene concentration of 8 µg/ml). The cells were centrifuged for 2 h at 1,000*g* at 32°C and incubated overnight at 37°C. The next day, supernatant was replaced with 2 ml of fresh complete RPMI and 48 h after transduction, antibiotic selection was performed throughout a period of at least 10 days by adding puromycin (1 μg/ml). The transduction of *JHDM1D-AS1* in THP1-MD2-CD14 cells was confirmed with RT-qPCR.

THP1-MD2-CD14 cells overexpressing *JHDM1D-AS1* transcripts or control cells (empty vector) in RPMI 1640 containing 10% FCS, penicillin (100 U/ml), and 2 mM L-glutamine were seeded at a density of 5 × 10^5^cells per well in a 48-well plate and stimulated with LPS (100 ng/ml) or medium control for 2 and 6 h.

### RNA Isolation and Sequencing

Total RNA was isolated from the following: (i) JHDM1D-AS1–deficient MDMs (n = 4) and control cells (n = 4), exposed to LPS (100 ng/ml) or not (medium control) for 6 h; and (ii) THP1-MD2-CD14 cells expressing ectopic *JHDM1D-AS1* (n = 4) or empty vector control (n = 4), treated with LPS (100 ng/ml) or medium control for 2 and 6 h. We used RNeasy mini kits (Qiagen, Hilden, Germany) according to the manufacturer’s instructions. RNA quality was assessed by bioanalysis (Agilent), with all samples having RNA integrity numbers > 7. Total RNA concentrations were determined by Qubit^®^ 2.0 Fluorometer (Life Technologies, Carlsbad, CA, USA). RNA sequencing libraries were prepared from 300 ng of total RNA using KAPA RNA HyperPrep with RiboErase (Roche) library kits and sequenced using the Illumina HiSeq4000 instrument (Illumina) to generate single reads (50 bp). Quality was assessed using FastQC [v0.11.5; Trimmomatic version 0.36 (Bolger et al., 2014) was used to trim Illumina adapters and poor-quality bases (trimmomatic parameters: leading = 3, trailing = 3, sliding window = 4:15, minimum length = 40)]. The remaining high-quality reads were used to align against the Genome Consortium human genome build 38 (GRCh38). Mapping was performed by HISAT2 version 2.1.0 (Kim et al., 2015) with parameters as default. Count data of the siRNA knockdown experiments were generated by means of the HTSeq method (Anders et al., 2015), whereas featureCounts (Liao et al., 2014) was used for overexpression experiments. Counts were subsequently analyzed using DESeq2 (Love et al., 2014) in the R statistical computing environment (R Core Team 2014. R: A language and environment for statistical computing. R Foundation for Statistical Computing, Vienna, Austria). Canonical signaling pathways were inferred using Ingenuity Pathway Analysis (QIAGEN bioinformatics), specifying human species and ingenuity database as reference. All other parameters were default. Statistically significant differences were defined by Benjamini–Hochberg adjusted probabilities < 0.05. Monocyte transcriptomes from healthy donors and patients with sepsis (Khan et al., 2020) were analyzed for proportions of cell subsets by means of a transcriptome deconvolution method, Absolute Immune Signal (ABIS), by applying the online Shiny application (https://github.com/giannimonaco/ABIS) (Monaco et al., 2019). Sequence libraries are publicly available through the National Center for Biotechnology Information (NCBI) gene expression omnibus (GEO) with accession identifier GSE201958.

### Public Gene Expression Data

Expression patterns of JHDM1D-AS1 were examined in an additional cohort of all-cause patients with sepsis (n = 156) relative to healthy subjects (n = 82) publicly available in the GEO NCBI database under accession number GSE134347 (Scicluna et al., 2020).

## Results

### *JHDM1D-AS1* Expression Is Induced in Primary Human Monocytes During Sepsis and After Exposure to TLR Ligands or Bacteria

**Figure 1:**
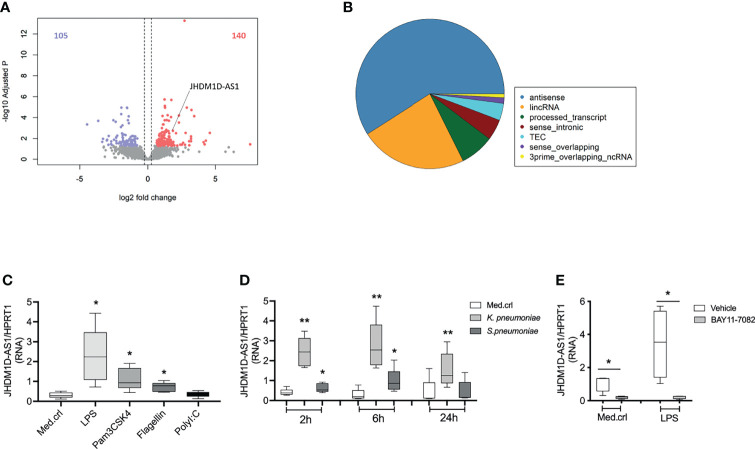
*JHDM1D-AS1* expression patterns in primary human monocytes during sepsis and after stimulation. **(A)** Volcano plot showing significantly altered long non-coding RNA (lncRNA) identified by RNA sequencing analysis of CD14^+^ monocytes purified from critically ill patients with sepsis due to community-acquired pneumonia (CAP), relative to healthy donors (n = 6). Red denotes elevated expression (adjusted p < 0.05, fold change ≥ 1.5); blue denotes reduced expression (adjusted p < 0.05, fold change ≤ −1.5). **(B)** Pie chart illustrating the distribution of lncRNA biotypes among significantly altered lncRNAs. **(C, D)** RT-qPCR analysis of *JHDM1D-AS1* expression in primary monocytes exposed to different Toll-like receptor (TLR) ligands for 2 h or challenged with heat-killed bacteria for 2, 6, and 24 h (n = 5). **(E)** Primary monocytes were pretreated (−30 min) with BAY11-7082 or DMSO (vehicle) followed by LPS stimulation for 2 h (n = 4). The expression levels of *JHDM1D-AS1* were quantified with RT-qPCR. The boxes extend from the 25th to 75th percentiles, the whiskers range from minimum to maximum, and the horizontal line indicates the median. Paired student’s t-tests were performed for the indicated comparisons. *p < 0.05; **p < 0.01.

To evaluate lncRNA expression in monocytes, we performed RNA sequencing in primary human blood monocytes (CD14^+^) obtained from patients with sepsis due to community-acquired pneumonia and healthy subjects (Khan et al., 2020). No differences were detected in monocyte subsets (classical, non-classical, or intermediate) by deconvolution of absolute immune signal (**Supplementary Figure 1A**). Relative to health, 245 lncRNAs were significantly altered in monocytes of patients with sepsis (adjusted p-value < 0.05; fold expression ≥ 1.2 or ≤ −1.2), representing 3.9% of all lncRNAs identified; 140 lncRNAs were elevated and 105 lncRNAs were reduced in monocytes of patients with sepsis (**Figure 1A**). A substantial proportion of altered lncRNAs belonged to the antisense RNA family (**Figure 1B**). Among these antisense RNAs, we identified the previously described *JHDM1D-AS1* (Kondo et al., 2017; Yao et al., 2019; Shi et al., 2019; Liu et al., 2020), which is physically located on chromosome 7 (140,177,261–140,179,640 bp). Expression patterns of *JHDM1D-AS1* antisense RNA were also significantly higher in whole-blood transcriptomes obtained from publicly available data of all-cause patients with sepsis (n = 156) relative to healthy donors (n = 82) (**Supplementary Figure 1B**) (Scicluna et al., 2020). Next, we used RT-qPCR to examine the inducibility of *JHMD1D-AS1* in primary human monocytes obtained from healthy donors, stimulated with LPS (TLR4 agonist), Pam3CysSerLys4 lipopeptide (PAM3CSK4; TLR1/2 agonist), flagellin (TLR5 agonist), or polyinosinic:polycytidylic acid [poly (I:C); TLR3 agonist]. *JHDM1D-AS1* expression was significantly induced by LPS, Pam3CSK4, and flagellin, but not poly(I:C) (**Figure 1C**), suggesting that MyD88 (adapter protein for all TLRs except TLR3) but not TRIF (TIR domain–containing adapter-inducing interferon-β; adaptor protein for TLR3) drives *JHDM1D-AS1* expression. Moreover, expression of *JHDM1D-AS1* was significantly induced in monocytes after stimulation with clinically relevant pathogens, *S. pneumoniae* (gram-positive) and *K. pneumoniae* (gram-negative), which peaked after 6 h (**Figure 1D**). Expression kinetics of *JHDM1D-AS1* after stimulation with these bacteria closely followed mRNA expression and secreted protein kinetics of TNF and IL-6, but not IL-10 (**Supplementary Figures 2A–E**). The expression kinetics of *JHDM1D-AS1* after exposure to pathogen lysates was corroborated by stimulations with LPS (**Supplementary Figures 3A–G**). These results indicate that *JHDM1D-AS1* expression is induced during sepsis and by various TLR ligands in primary human monocytes.

TLR activation triggers MyD88-dependent signaling pathways that culminate in the activation of NF-κB, a critical transcription factor that controls expression of multiple immune response genes. To evaluate the impact of NF-κB on the inducibility of *JHDM1D-AS1* expression, we pretreated (−30 min) monocytes from healthy donors with BAY11-7082, an irreversible inhibitor of the IkappaB kinase kinases at 10 μM (Malladi et al., 2004; Atianand et al., 2016), followed by LPS stimulation for 2 h. LPS-induced secretion of TNF and expression of *JHDM1D-AS1* were significantly reduced by BAY11-7082 (**Figure 1E** and **Supplementary Figure 1C**), indicating that the inducibility of *JHDM1D-AS1* expression depends, at least in part, on NF-kB activation.

### JHDM1D-AS1 Deficiency in Macrophages Results in an Enhanced Inflammatory Response

**Figure 2:**
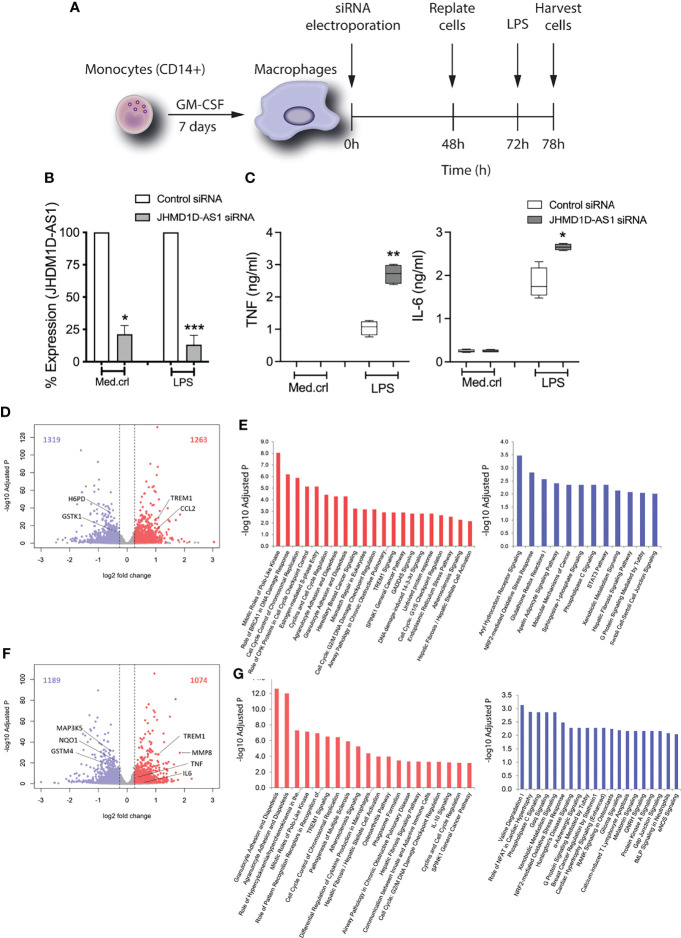
JHDM1D-AS1 deficiency leads to increased pro-inflammatory gene responses in human monocyte-derived macrophages upon LPS exposure. The expression of *JHDM1D-AS1* was modified in monocyte-derived macrophages using siRNA electroporation. **(A)** Schematic overview of the experimental setup. **(B)** *JHDM1D-AS1* expression (analyzed by RT-qPCR) in JHDM1D-AS1–deficient or control cells, showing a 70%–80% reduction in JHDM1D-AS1 levels in the former. **(C)** The secreted levels of TNF and IL-6 were determined in supernatants using ELISA. Error bars indicate SD **(B)**, whereas the boxes **(C)** extend from the 25th to 75th percentiles, the whiskers range from minimum to maximum, and the horizontal line indicates the median. Unpaired student’s t-test on raw data **(B)** and paired student’s t-tests **(C)** were performed respectively for the indicated comparisons. *p < 0.05; **p < 0.01; ***p < 0.001. **(D)** Volcano plot illustrating basal levels of gene expression significantly altered due to JHDM1D-AS1 deficiency (relative to control cells). Red denotes elevated expression (adjusted p < 0.05, fold change ≥ 1.5); blue denotes reduced expression (adjusted p < 0.05, fold change ≤ −1.5). **(E)** Ingenuity pathway analysis of the significantly altered RNA transcripts at baseline. **(F)** Volcano plot depicting significantly altered genes, and **(G)** ingenuity pathways analysis of those significant transcripts in JHDM1D-AS1–deficient cells, relative to control cells, after exposure to LPS (100 ng/ml). Red denotes elevated expression (adjusted p < 0.05, fold change ≥ 1.5); blue denotes reduced expression (adjusted p < 0.05, fold change ≤ −1.5).

We next set out to evaluate the regulatory potential of JHDM1D-AS1 on the inflammatory response. To address this aim, we used siRNA electroporation to introduce a pool of four JHDM1D-AS1–specific siRNA or control siRNA in primary human macrophages (**Figure 2A**). MDMs, in conformity with monocytes, also produced JHDM1D-AS1 in a temporal specific pattern after LPS stimulation (**Supplementary Figure 4A**). Silencing of JHM1D-AS1 by RNA interference resulted in ~80% knockdown of JHDM1D-AS1 expression, both basal and after stimulation with LPS for 6 h relative to control cells (**Figure 2B**).

JHDM1D-AS1*–*deficient cells secreted significantly more TNF and IL-6 after LPS stimulation (**Figure 2C**). This observation was also confirmed in more biological replicates (**Supplementary Figures 4B,C**). To evaluate the influence of JHDM1D-AS1 on the LPS-inducible transcriptome, we performed RNA sequencing of JHDM1D-AS1*–*deficient and control cells, at basal conditions and after a 6-h exposure to LPS. Relative to control cells, basal levels of 2,582 RNA transcripts were significantly altered (adjusted p-value < 0.05; fold expression ≥ 1.2 or ≤ −1.2) in JHDM1D-AS1*–*deficient cells (1,263 upregulated and 1,319 downregulated) (**Figure 2D**). Ingenuity pathway analysis of upregulated genes revealed an association with various cell growth, cell cycle, DNA damage, and inflammatory response pathways, including mitotic roles of polo-like kinase, role of BRCA1 in DNA damage response, agranulocyte adhesion and aiapedesis (including *CXCL2*, *CXCL3*, *CXCL8*, and *IL1RN*), and TREM1 signaling (with *TREM1*, *IL6*, *CCL2*, and *CCL3*) (**Figure 2E**). Downregulated genes were associated with mainly transcription factor and metabolic pathways, including the aryl hydrocarbon receptor signaling (with *ALDH1A1*, *ALDH2*, *ESR1*, and *GSTK1*), STAT3 pathway (having *SOCS1*, *TGFA*, *CXCR1*, *IL10RA*, and *IL1R1*), and pentose phosphate pathway (including *G6PD*, *H6PD*, and *PGD*). These findings suggest that basal expression of *JHDM1D-AS1* may influence cell growth and homeostasis. In line with these observations, *JHDM1D-AS1* was shown by others to promote tumor cell growth and metastasis (Kondo et al., 2017; Yao et al., 2019). Comparing the transcriptomes of LPS-exposed JHDM1D-AS1*–*deficient cells to LPS-exposed control cells, unmasked 2,263 significantly altered transcripts (adjusted p-value < 0.05; fold expression ≥ 1.2 or ≤ −1.2) with 1,074 upregulated and 1,189 downregulated RNA transcripts (**Figure 2F**). Upregulated genes were associated with primarily cell mobility, cell cycle, cell development, and inflammatory pathways that included granulocyte adhesion and diapedesis, cell cycle control of chromosomal replication, differential regulation of cytokine production in macrophages, TREM1 signaling, and IL-10 signaling (**Figure 2G**). Among the upregulated genes were several well-known inflammatory mediators such as *CXCL2*, *CXCL8*, *CCL3L1*, *IL1RN*, *TREM1*, *TNF*, and *IL6.* Downregulated genes were associated with transcription factor and metabolic and oxidative stress response pathways, including NRF2-mediated oxidative stress responses. Considering the differences that we observed in basal conditions, we fit a linear model that also accounted for those baseline differences. In this way, we identified 29 significantly altered RNA transcripts in LPS-stimulated JHDM1D-AS1*–*deficient cells (**Supplementary Figure 4D**). Altogether, these findings suggest that *JHDM1D-AS1* plays a dual role, both at a basal cell level and upon TLR-dependent cell activation. JHDM1D-AS1 deficiency at a basal level influenced gene expression pathways attuned to cell development and hemostasis, whereas LPS exposure resulted in an enhanced inflammatory response.

### *JHDM1D-AS1* Overexpression Dampens the Inflammatory Response in Pro-Monocytic Cells

**Figure 3:**
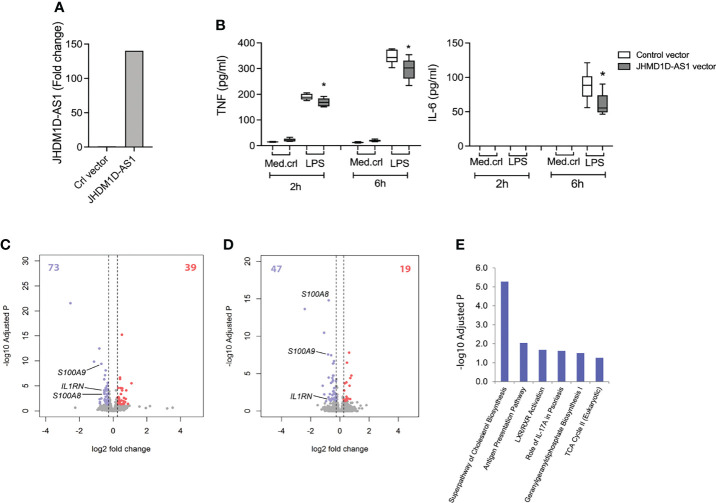
Overexpression of JHDM1D-AS1 in THP-1-MD2-CD14 pro-monocytic cells leading to reduced inflammatory responses upon LPS stimulation. **(A)** RT-qPCR analysis of *JHDM1D-AS1* levels in THP1-MD2-CD14 cells expressing ectopic JHDM1D-AS1 or empty vector control. **(B)** Boxplots depicting secreted levels of TNF and IL-6 in supernatants of THP1-MD2-CD14 cells expressing ectopic JHDM1D-AS1 or empty vector (control), exposed to LPS (100 ng/ml) or medium control for 2 or 6 h, quantified by ELISA. The boxes extend from the 25th to 75th percentiles, the whiskers range from minimum to maximum, and the horizontal line indicates the median. Unpaired student’s t-tests were performed for the indicated comparisons with *p < 0.05. **(C, D)** Volcano plots illustrating significantly altered RNA transcripts after LPS stimulation for **(C)** 2 h and **(D)** 6 h. Red denotes elevated expression (adjusted p < 0.05, fold change ≥ 1.5); blue denotes reduced expression (adjusted p < 0.05, fold change ≤ −1.5). **(E)** Significantly downregulated genes after 6-h LPS exposure were associated to Ingenuity canonical signaling pathways.

Next, we performed “gain-of-function” studies by overexpressing JHDM1D-AS1 using retroviral transduction of a *JHDM1D-AS1* overexpression vector or a control vector in a THP1-MD2-CD14 pro-monocytic cell line. THP1-MD2-CD14 cells are a suitable model system because the expression of *JHDM1D-AS1*, both at baseline and upon LPS stimulation, is low compared with primary monocytes and macrophages (**Supplementary Figure 5A**). This approach led to a 150-fold increase of *JHDM1D-AS1* expression in THP1-MD2-CD14 cells relative to controls (**Figure 3A**). After exposure to LPS for 2 or 6 h, THP1-MD2-CD14 cells overexpressing *JHDM1D-AS1* secreted lower levels of TNF and IL-6 as compared with control cells (**Figure 3B**). RNA sequencing and differential expression analysis showed minimal basal differences between *JHDM1D-AS1* overexpression and control cells (**Supplementary Figures 5B,C** **)**. After 2 h of LPS exposure, 112 RNA transcripts were significantly altered (39 upregulated and 73 downregulated) in *JHDM1D-AS1* overexpression cells compared with control cells (**Figure 3C**). Of note, inflammatory response genes that included *S100A8*, *S100A9*, *CCL3L1*, and *IL1RN* were significantly downregulated in *JHDM1D-AS1* overexpression cells. We observed similar patterns after 6 h of LPS exposure, with 19 upregulated and 47 downregulated RNA transcripts in *JHDM1D-AS1* overexpression cells relative to control cells (**Figure 3D**). Again, *S100A8*, *S100A9*, and *IL1RN* were significantly downregulated. Ingenuity pathway analysis revealed significant associations of downregulated genes in *JHDM1D-AS1* overexpression cells stimulated with LPS for 2 h. We found enrichment of genes involved in cholesterol biosynthesis, antigen presentation, LXR/RXR activation, IL-17A signaling, geranylgeranyl diphosphate biosynthesis I, and tricarboxylic acid cycle (**Figure 3E**). Therefore, *JHDM1D-AS1* overexpression in THP1-MD2-CD14 pro-monocytic cells resulted in a dampened inflammatory response after exposure to LPS.

## Discussion

Here, we have identified the antisense lncRNA JHDM1D-AS1 as a potential novel repressor of the inflammatory response in human monocytes and macrophages. *JHDM1D-AS1* expression was upregulated in circulatory monocytes obtained from critically ill patients with sepsis caused by community-acquired pneumonia. RNA sequencing analysis of JHDM1D-AS1–depleted macrophages showed a two-pronged effect: (1) basal expression of *JHDM1D-AS1* may influence cell development and homeostasis pathways, and (2) enhanced inflammatory responses to LPS. Moreover, and in agreement with our loss-of-function studies, overexpression experiments in THP1-MD2-CD14 pro-monocytic cells showed dampened inflammatory responses after exposure to LPS. Our results provide new and compelling indications that JHDM1D-AS1 may regulate pro-inflammatory gene expression during the primary phases of infection.

A recent study provided evidence for a role of *JHDM1D-AS1* in rat microglia cells during neuronal injury (Liu et al., 2020). Interestingly, *JHDM1D-AS1* expression was downregulated in microglial cells upon LPS-induced TLR4 activation, whereas overexpression of *JHDM1D-AS1* suppressed inflammatory responses, potentially by targeting NF-ĸB-activation through the miR-101-3p/DUSP1 pathway. Whereas data on *JHDM1D-AS1* expression contrast with our results in primary human monocytes and MDMs, the latter observation is in line with our gain-of-function data implicating *JHDM1D-AS1* as a potential repressor of inflammatory responses. Microglial cells are the primary immune cells of the central nervous system and display many similarities with macrophages, for example, the ability to phagocytose and to initiate an inflammatory response (Xu et al., 2016). Nevertheless, there is considerable heterogeneity within the group of tissue macrophages with distinct transcriptomes and epigenomes consistent with their origin (Prinz et al., 2019), which can explain the differences in *JHDM1D-AS1* expression between human monocytes and macrophages, to those of rat microglia. Another possible explanation is the interspecies differences because murine cell lines were used for mechanistic studies by Liu and colleagues (Liu et al., 2020) compared with primary human cells used in ours. Whereas our study does not decipher the exact molecular mechanism by which *JHDM1D-AS1* impacts inflammatory responses in human monocytes and macrophages, it likely differs from the mechanism proposed by Liu et al. in microglia (Liu et al., 2020), *via* a DUSP1 and miR-101-3p axis. First, the expression pattern of both *JHDM1D-AS1* and DUSP-1 differed, and, second, miR-101-3P was not detected in our experimental setup. Altogether, this suggests that *JHDM1D-AS1* may exert species- or context-dependent functions depending on cell type and tissue localization.

Previous reports have shown that *JHDM1D-AS1* was upregulated in various cancers and, notably, affected tumor growth, angiogenesis, and apoptosis (Kondo et al., 2017; Yao et al., 2019; Shi et al., 2019). Those observations are in line with our loss-of-function findings in MDMs, showing that basal expression of *JHDM1D-AS1* influenced genes involved in cell development, cell cycle, and DNA damage repair. Moreover, genome-wide expression profiling of *JHDM1D-AS1* expressing pancreatic cancer cells in mouse tumor xenografts showed increased expression of inflammatory response genes *S100a8* (or *Mrp8*) and *S100a9* (or *Mrp14*) (Kondo et al., 2017). Our gain-of-function analysis also showed altered expression patterns of *S100A8* and *S100A9*, which are endogenous agonists of TLR4 and—as a heterodimer—play a key role in LPS induced shock *in vivo* (Achouiti et al., 2012). Another interesting observation is that *CCL3L1*, an isoform of macrophage inflammatory protein-1α, was upregulated in JHDM1D-AS1–deficient cells, whereas downregulated in THP1-MD2 cells overexpressing JHDM1D-AS1 after LPS exposure. Earlier studies have demonstrated that *CCL3L1* was induced in activated macrophages and participates in the early innate immune response (Menten et al., 1999), as well as exhibiting chemotactic activity for lymphocytes and monocytes (Menten et al., 2002). Whereas our observations, and those of others, further support a role for *JHDM1D-AS1* in the regulation of innate immune response genes, altogether, our data provide more far-reaching insights into antisense RNA biology.

Antisense transcription was initially considered as “transcriptional noise”, with no functional connotations. However, it is being increasingly recognized as a key factor in gene regulation by coordinating gene expression whether in *cis* or in *trans* (Pelechano and Steinmetz, 2013). Thus, antisense RNA can regulate gene expression in multiple ways, from influencing chromatin accessibility and methylation to the stability of transcribed mRNAs. By virtue of their highly specific regulatory potential, antisense RNAs hold much promise as therapeutic drug targets, particularly because advances in RNA biology have largely addressed outstanding challenges in RNA therapeutics. Chemical modifications of RNAs, such as addition of a phosphorothioate bonds, as well as nucleoside modifications that include N6-methyladenosine, have largely addressed the overarching challenges of RNA degradation and activation of inflammatory responses, respectively (Karikó et al., 2005; Kole et al., 2012). We envisage that our findings could constitute a benchmark in investigating RNA therapeutics in the context of sepsis, defined as a syndrome of life-threatening organ dysfunction caused by a dysregulated host response to infection (Singer et al., 2016). However, further studies are necessary to establish how JHDM1D-AS1 is regulated *in vivo* during sepsis, whether expression is influenced by divergent infectious etiologies, and its role in the immunopathology of sepsis. Whether delivery of a stable form of JHDM1D-AS1 to immune cells *in vitro* or to *in vivo* models of acute inflammation and/or sepsis can dampen deleterious inflammatory responses certainly warrants exploration.

In summary, our findings provide evidence that *JHDM1D-AS1* expression is upregulated in monocytes of patients with sepsis, induced upon TLR-activation, and acts as a repressor of the inflammatory response in monocytes/macrophages. The insight obtained from this study further advances our understanding of antisense RNAs and their physiological role in host defense and inflammation.

## Data Availability Statement

The datasets presented in this study can be found in online repositories. The names of the repository/repositories and accession number(s) can be found below: https://www.ncbi.nlm.nih.gov/, GSE201958.

## Ethics Statement

The Medical Ethics Committee of the Academic Medical Center approved the study (IRB no. 10-056C), and written informed consent was obtained from all patients (or legal representative) and healthy controls for transcriptomics or in vitro stimulation assays.

## Author Contributions

EM performed the experiments, analyzed the data, and wrote the manuscript. HK analyzed the RNA sequencing data and contributed to the interpretation of the data. MS, MM, HM, and NO performed parts of the experiments. TG, AD, and CV supplied reagents and contributed to the interpretation of the data and revised the manuscript for important intellectual content. TP supervised the project and revised the manuscript for important intellectual content. BS supervised the project, analyzed the RNA sequencing data, contributed to the interpretation of the data, and wrote the manuscript. All authors read and approved the final version of the manuscript.

## Funding

EM is funded by Wenner-Gren Foundations (FT2020-0003) and Stiftelsen P E Lindahls stipendiefond Medicine (LM20170016).

## Conflict of Interest

The authors declare that the research was conducted in the absence of any commercial or financial relationships that could be construed as a potential conflict of interest.

## Publisher’s Note

All claims expressed in this article are solely those of the authors and do not necessarily represent those of their affiliated organizations, or those of the publisher, the editors and the reviewers. Any product that may be evaluated in this article, or claim that may be made by its manufacturer, is not guaranteed or endorsed by the publisher.
