# Supplementary material for: The Long Non-Coding Antisense RNA JHDM1D-AS1 Regulates Inflammatory Responses in Human Monocytes
Source: Front Cell Infect Microbiol. 2022 Jul 12;12:934313. doi: 10.3389/fcimb.2022.934313 (PMC9315269; doi:10.3389/fcimb.2022.934313)
Supplement: Supplementary file 1 [file DataSheet_1.pdf]

# *Supplementary Material*

## Supplementary Figure

Supplementary Figure 1 Malmström et al

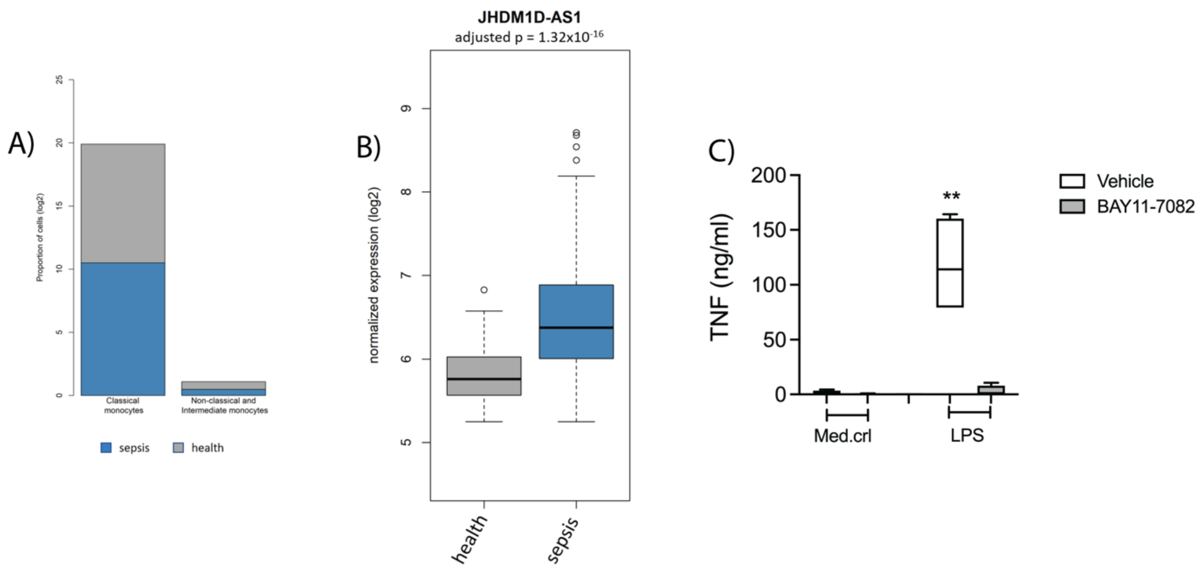


**Supplementary Figure 1. (A)** Monocyte transcriptomes from healthy donors and sepsis patients were analyzed for proportions of monocyte cell subsets by means of a transcriptome deconvolution method, Absolute Immune Signal (ABIS). No differences were detected in predicted monocyte subsets between sepsis patients and healthy donors, which showed a preponderance for classical monocytes. **(B)** Expression patterns of *JHDM1D-AS1* were examined in an additional cohort of all-cause sepsis patients (n=156) relative to healthy subjects (n=82). Data shows that the expression of *JHDM1D-AS1* was significantly higher in sepsis patients relative to health. **(C)** Primary monocytes were pretreated (-30 min) with BAY11-7082 or DMSO (vehicle) followed by LPS stimulation for 2 hours (4 replicates). The protein levels of secreted TNF were quantified using ELISA. The boxes extend from the 25th to 75th percentiles, the whiskers range from minimum to maximum and the horizontal line indicate the median. Unpaired student's t tests were performed for the indicated comparisons. $^{**}p < 0.01$.

Supplementary Figure 2 Malmström et al

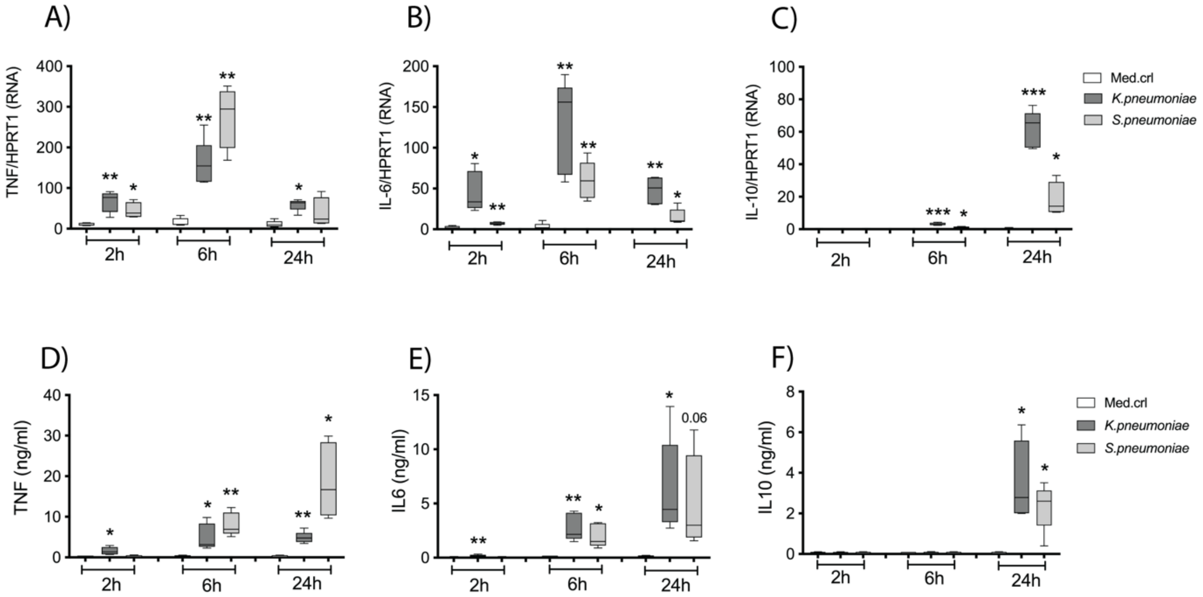


**Supplementary Figure 2. (A-C)** RT-qPCR analysis was performed to determine mRNA levels of cytokine genes in primary monocytes exposed to heat-killed *Streptococcus (S.)pneumoniae*, *Klebsiella (K.)pneumoniae*, or medium control, for 2, 6 and 24 hours (n=5 each). **(D-F)** Levels of secreted cytokines (TNF, IL-6 and IL-10) in the supernatants of challenged monocytes quantified by ELISA. The boxes extend from the 25th to 75th percentiles, the whiskers range from minimum to maximum and the horizontal line indicate the median. Paired student's t tests were performed for the indicated comparisons, relative to medium controls. *p < 0.05; **p < 0.01 and ***p < 0.001.

Supplementary Figure 3 Malmström et al

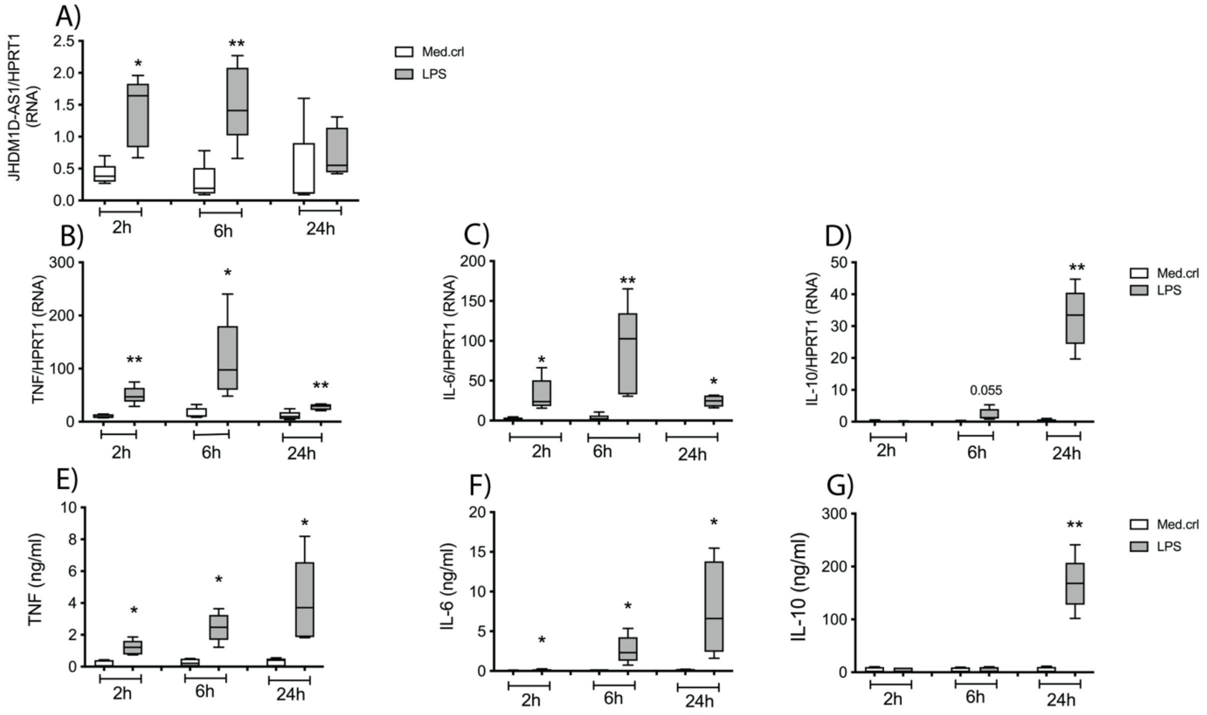


**Supplementary Figure 3. (A)** The RNA levels of *JHDM1D-AS1* were determined in monocytes exposed to 100 ng/ml LPS or medium control using RT-qPCR (n=5). **(B-D)** RT-qPCR analysis was performed to determine mRNA levels of cytokine genes in primary monocytes exposed to 100 ng/ml LPS or medium control, for 2, 6 and 24 hours (n=5 each). **(E-G)** Levels of secreted cytokines (TNF, IL-6 and IL-10) in the supernatants of challenged monocytes quantified by ELISA. The boxes extend from the 25th to 75th percentiles, the whiskers range from minimum to maximum and the horizontal line indicate the median. Paired student's t tests were performed for the indicated comparisons, relative to medium controls. *p < 0.05 and **p < 0.01

Supplementary Figure 4 Malmström et al

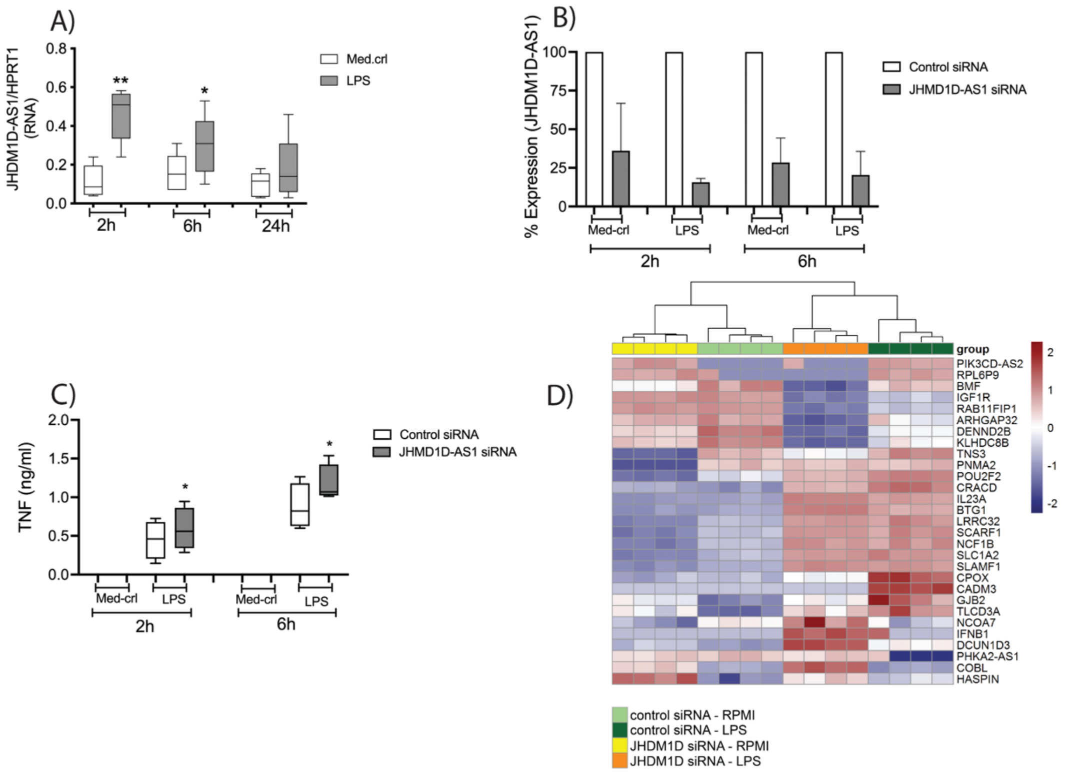


**Supplementary Figure 4. (A)** RT-qPCR analysis of JHDM1D-AS1 in monocyte-derived macrophages treated with 100 ng/ml LPS or medium control for 2, 6 and 24 hours (n=5). **(B)** Cells rendered deficient for JHDM1D-AS1 by small inhibitory RNA interference (siRNA) and control cells were exposed to 100 ng/ml LPS or medium control for 2 and 6 hours. JHDM1D-AS1 RNA levels was determined using RT-qPCR (n=4). Error bars indicate SD. **(C)** The protein levels of TNF secreted from control cells or JHDM1D-AS1 deficient cells were quantified with ELISA. Boxes extend from the 25th to 75th percentiles, whiskers range from minimum to maximum and the horizontal line indicate the median **(A and C)**. Paired student's t tests were performed for the indicated comparisons. *p < 0.05; **p < 0.01. **(D)** Heatmap representation of 29 significantly altered RNA transcripts in LPS-treated JHDM1D-AS1 deficient cells identified after fitting RNA-sequencing data in a linear model adjusting for the basal effect of JHDM1D-AS1 deficiency.

Supplementary Figure 5 Malmström et al

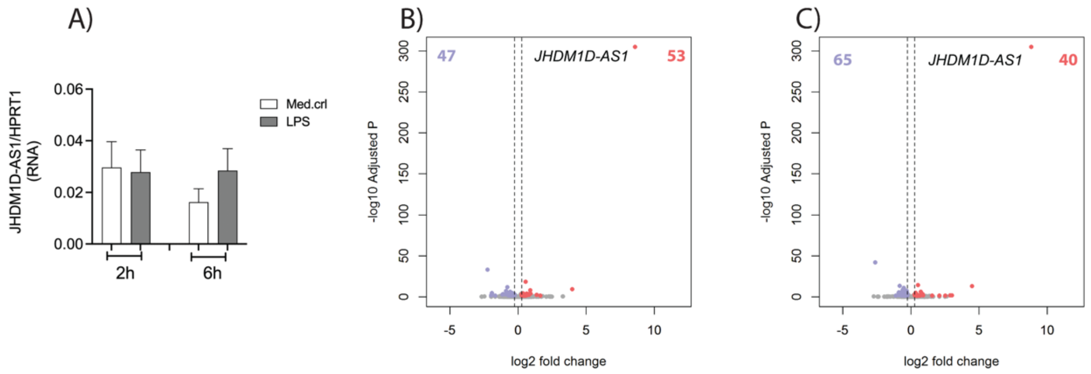


**Supplementary Figure 5.** (**A**) Bar plot depicting the levels of JHDM1D-AS1 RNA in THP1-MD2-CD14 cells stimulated with 100 ng/ml LPS or medium control for 2 and 6 hours, quantified with RT-qPCR. HPRT1 was used as endogenous control. Error bars indicate SD. **(B** and **C)** Retroviral transduction was used to ectopically overexpress JHDM1D-AS1 in THP1-MD2-CD14 cells. RNA-sequencing was performed in THP1-MD2-CD14 cells overexpressing JHDM1D-AS1, or control cells, were treated with 100ng/ml LPS or medium control for 2 and 6 hours. Data are depicted as volcano plots illustrating significantly altered RNA transcripts between THP1-MD2-CD14 cells overexpressing JHDM1D-AS1 relative to control cells at basal level, i.e., not LPS exposed, after (**B**) 2 hours, and (**C**) 6 hours. Red denotes elevated expression (adjusted $p < 0.05$, fold change $\geq 1.5$); blue denotes reduced expression (adjusted $p < 0.05$, fold change $\leq -1.5$).

**Supplementary table 1**

| Gene | Direction | Sequence |
|---|---|---|
| TNF | Fwd | CAGGGACCTCTCTCTAATCAGC |
| | Rev | GCTGGTTATCTCTCAGCTCCAC |
| IL-6 | Fwd | AACATGTGTGAAAGCAGCAAAG |
| | Rev | CTCTCAAATCTGTTCTGGAGGT |
| IL-10 | Fwd | GATCCAGTTTTACCTGGAGGAG |
| | Rev | TTAAAGGCATTCTTCACCTGCT |
| HPRT1 | Fwd | GGATTTGAAATTCCAGACAAGTTT |
| | Rev | GCGATATCAATAGGACTCCAG |
| JHDM1D-AS1 | Fwd | TGTCTCAATTCTGCAACTATTCG |
| | Rev | CAAGCTTAATCAGTGTGTGGTCA |

**Supplementary table 1.** Primers used for RT-qPCR
